# Supplementary material for: The Effect of Neurodynamic Techniques on the Dispersion of Intraneural Edema: A Systematic Review with Meta-Analysis
Source: Int J Environ Res Public Health. 2022 Nov 4;19(21):14472. doi: 10.3390/ijerph192114472 (PMC9655711; doi:10.3390/ijerph192114472)
Supplement: Supplementary file 1 [file ijerph-19-14472-s001.zip › ijerph-1940926-supplementary.pdf]

Supplementary Materials File

LEGEND

Table S1. Full search strategies for all databases.

Table S2. Studies excluded after full text read with the reason for exclusion.

Figure S1. Risk of bias of RCTs (RoB 2.0).

Figure S2. Risk of bias of nonrandomized studies (ROBINS-I).

Figure S3. Forest plot of sensitivity analysis.

Figure S4. Funnel Plot

Table S1. Search strategy for each database.

Date: 12 February 2022**.**

MEDLINE (via Pubmed)

("nerve therapy" OR "nerve treatment" OR "neural treatment" OR neurodynamic OR neurodynamics OR "manual therapy" OR "nerve stretch" OR "nerve tension" OR "neural tension" OR "Nerve mobilization" OR "neural mobilization" OR "Nerve glide" OR "nerve gliding" OR "neural glide" OR "neural gliding" OR "nerve gliding exercises" OR neuromobilization OR "neuromobilization maneuver" OR "neurodynamic techniques") AND ("fluid dispersion" OR "dye spread" OR "intraneural edema" OR "intraneural oedema")

Results: 6

Scopus

TITLE-ABS-KEY (("nerve therapy" OR "nerve treatment" OR "neural treatment" OR neurodynamic OR neurodynamics OR "manual therapy" OR "nerve stretch" OR "nerve tension" OR "neural tension" OR "Nerve mobilization" OR "neural mobilization" OR "Nerve glide" OR "nerve gliding" OR "neural glide" OR "neural gliding" OR "nerve gliding exercises" OR neuromobilization OR "neuromobilization maneuver" OR "neurodynamic techniques")) **AND TITLE-ABS-KEY ((**"fluid dispersion" OR "dye spread" OR "intraneural edema" OR "intraneural oedema")

Results: 7

Web Of Science (All databases)

("nerve therapy" OR "nerve treatment" OR "neural treatment" OR neurodynamic OR neurodynamics OR "manual therapy" OR "nerve stretch" OR "nerve tension" OR "neural tension" OR "Nerve mobilization" OR "neural mobilization" OR "Nerve glide" OR "nerve gliding" OR "neural glide" OR "neural gliding" OR "nerve gliding exercises" OR neuromobilization OR "neuromobilization maneuver" OR "neurodynamic techniques") AND ("fluid dispersion" OR "dye spread" OR "intraneural edema" OR "intraneural oedema")

Results: 18

Cochrane Database

("nerve therapy" OR "nerve treatment" OR "neural treatment" OR neurodynamic OR neurodynamics OR "manual therapy" OR "nerve stretch" OR "nerve tension" OR "neural tension" OR "Nerve mobilization" OR "neural mobilization" OR "Nerve glide" OR "nerve gliding" OR "neural glide" OR "neural gliding" OR "nerve gliding exercises" OR neuromobilization OR "neuromobilization maneuver" OR "neurodynamic techniques") AND ("fluid dispersion" OR "dye spread" OR "intraneural edema" OR "intraneural oedema")

Results: 4

TOTAL results: 35

Results after duplicated removed: 20 (with Mendeley desktop find and merge duplicated tool).

Table S2. Studies excluded after full text read with the reason for exclusion [30].

**Reference** (Author and year)	**Reason for exclusion**
Schmid et al, 2012	Study in humans

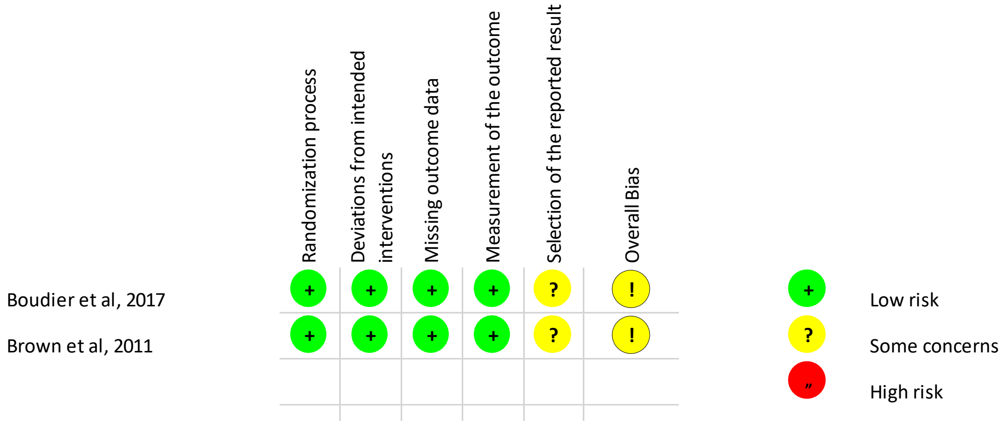

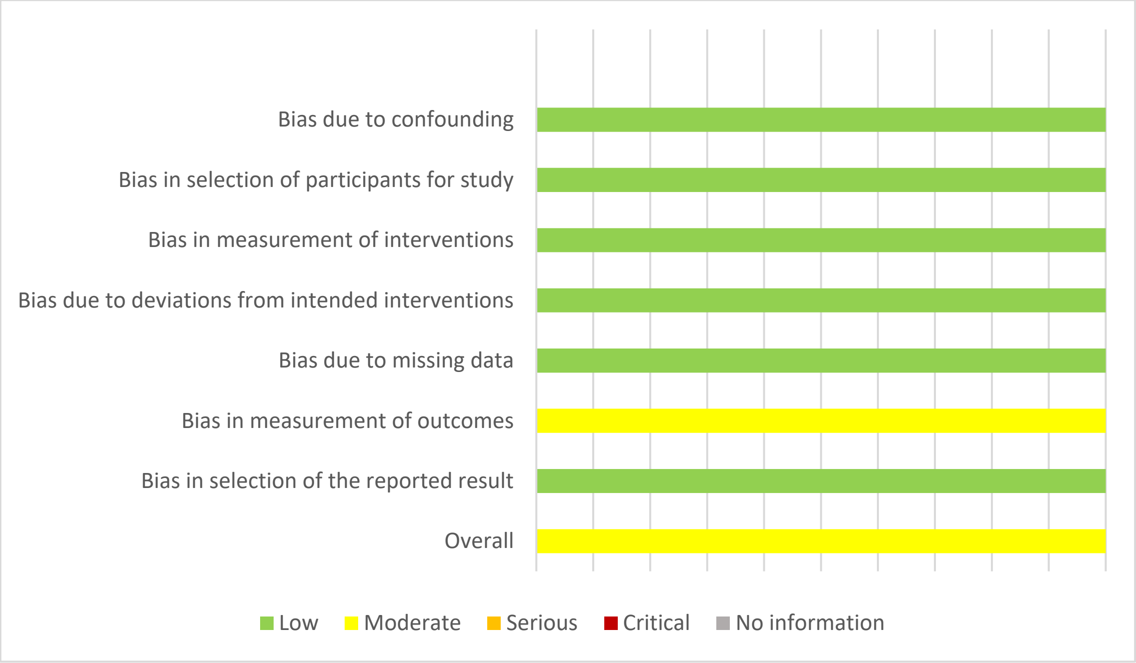

Figure S1. Risk of bias of RCTs (RoB 2.0) [28,29].

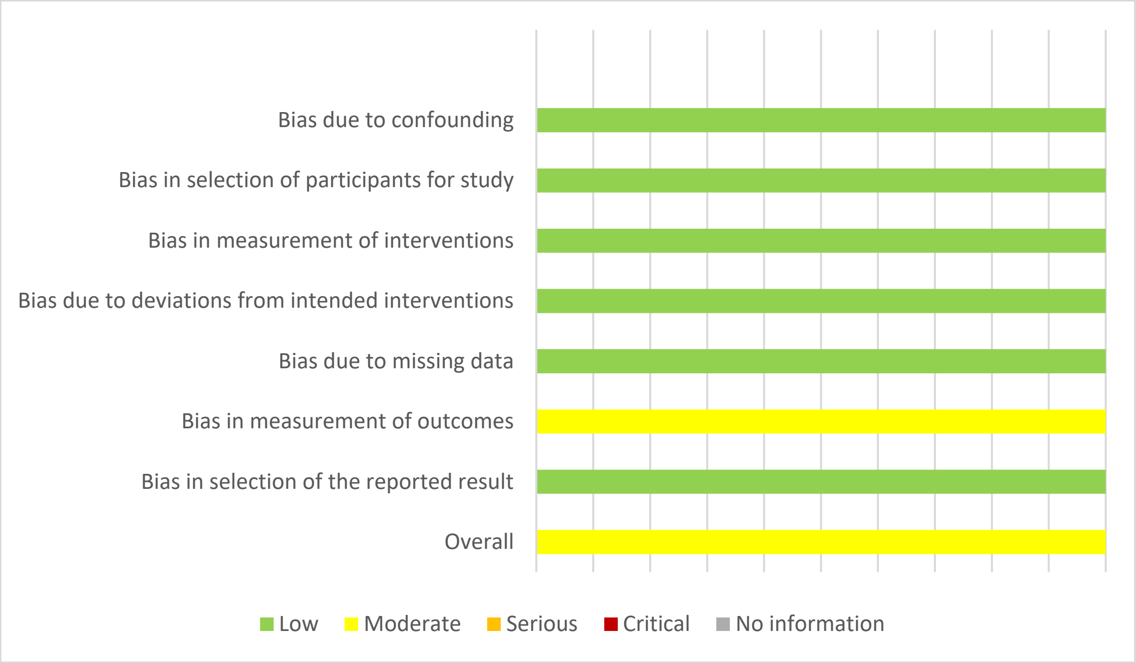

Figure S2. Risk of bias of nonrandomized studies (ROBINS-I)

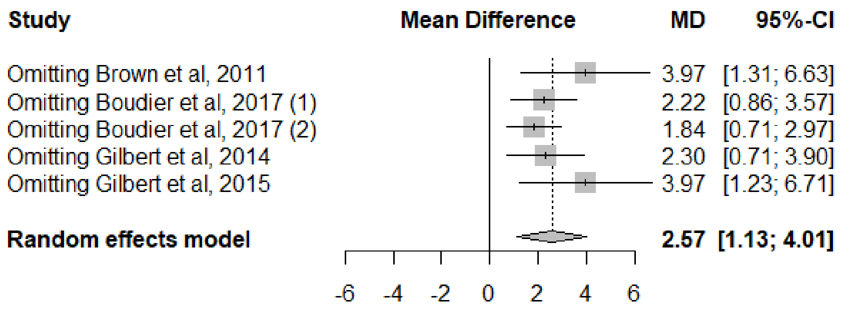

Figure S3. Forest plot of sensitivity analysis [26–29].

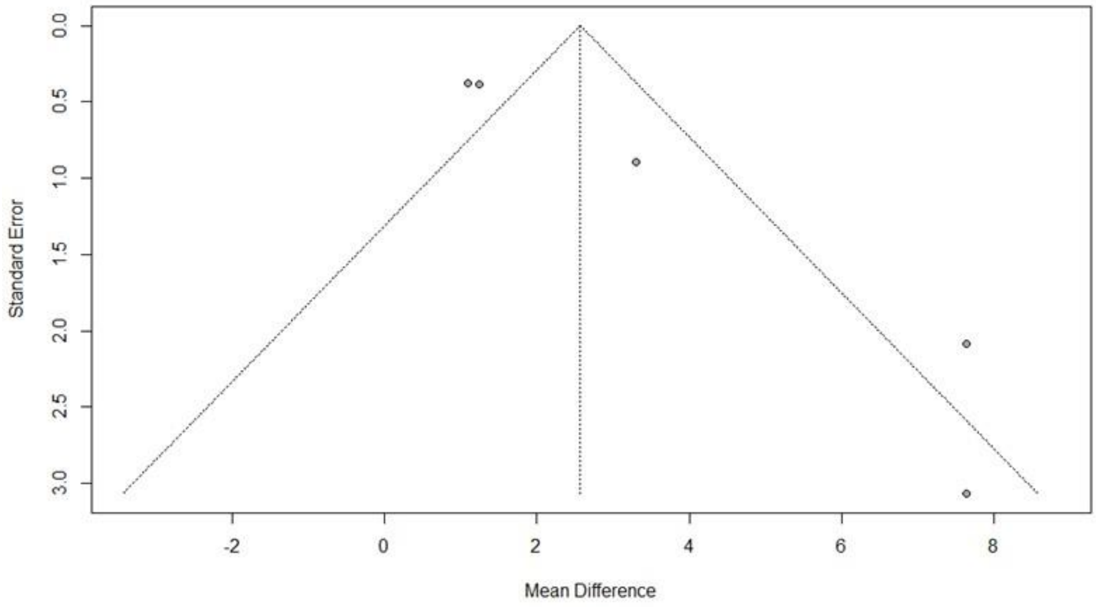

Figure S4. Funnel Plot.
